# Cesarean scar pregnancy with expectant management

**DOI:** 10.1111/jog.15258

**Published:** 2022-04-05

**Authors:** Liye Fu, Yingchun Luo, Jinbai Huang

**Affiliations:** ^1^ Department of Medical Imaging Medical College of Yangtze University Jingzhou Hubei China; ^2^ Department of Ultrasonography Changsha Hospital for Maternal and Child Health Care Changsha Hunan China; ^3^ Department of Ultrasonography Maternal and Child Health Hospital of Hunan Province Changsha Hunan China; ^4^ Department of Medical Imaging The First Affiliated Hospital of Yangtze University Jingzhou Hubei China

**Keywords:** cesarean scar pregnancy, expectant management, intraoperative blood loss, morbidly adherent placenta, ultrasound examination

## Abstract

**Aim:**

This study aimed to ascertain whether the lower anterior myometrial thickness (MT) between the bladder and the gestational sac in early pregnancy can be used to predict clinical outcomes in women with cesarean scar pregnancy (CSP) after expectant management.

**Methods:**

We retrospectively analyzed the clinical data and early pregnancy ultrasound images of 21 patients who received expectant management for CSP. Among them, 11 patients with serious complications during pregnancy, such as intraoperative blood loss ≥1000 mL or with severe forms of morbidly adherent placenta (MAP; placenta increta or placenta percreta), were assigned to group A. The remaining 10 patients without serious complications during pregnancy were assigned to group B. The difference in MT between groups A and B was analyzed using nonparametric Mann–Whitney *U* test.

**Results:**

There was a statistically significant difference in MT between the groups (*U* = 20.000, *p* = 0.013). The area under the receiver operating characteristics (ROC) curve was 0.818, and the optimal cut‐off value for MT was 3.3 mm.

**Conclusion:**

Lower anterior MT around the gestational sac was correlated with severe complications, such as massive intraoperative bleeding or severe forms of MAP in patients with CSP.

## Introduction

Cesarean scar pregnancy (CSP) is a special type of ectopic pregnancy in which the gestational sac is implanted on the scar of the uterine incision from the previous cesarean section.^1^ CSP is one of the long‐term complications of cesarean section, with an incidence rate of 1:2656–1:1800 among all pregnancies.^2^ With the introduction of China's two‐child policy and the increase in cesarean section rate, the CSP incidence rate has gradually increased. Studies^3^, ^4^, ^5^ have found the absence of decidua and the implantation of villus tissue implanted at the scar. The hypoxic environment stimulates the trophoblast cells to invade deep into the muscle layer, thus inducing morbidly adherent placenta (MAP). CSP and MAP are believed to have the same histopathological characteristics. Based on the degree to which the placental villus tissue invades the muscular layer, there are three types of MAP.^6^, ^7^ When villous tissue penetrates the decidual baseline to reach the muscular layer, it is called placenta accreta. When villous tissue invades the deep muscular layer, it is called placenta increta. When villous tissue penetrates the muscular layer to reach the serosal layer, and, in some cases, implants into parauterine tissues, such as the bladder and urethra, it is called placenta percreta. Pregnancy outcomes can be affected by different types of placental implantation in CSP. In some patients with severe forms of MAP, a hysterectomy is necessary to prevent the death of the mother.^6^, ^8^

Since CSP is prone to severe complications such as massive bleeding, MAP, and uterine rupture during the middle and late pregnancy, it is usually recommended to terminate pregnancy early once CSP is diagnosed clinically.^2^ However, some patients, especially those with difficulties in getting pregnant, refuse to terminate the pregnancy, and choose expectant management instead. In clinical practice, some pregnant women are lucky enough to give birth to live babies.^9^, ^10^, ^11^ At present, there are no standardized evaluation criteria and guidance indicators, so it is very important to find reliable indicators in early pregnancy that can guide clinical expectant treatment for CSP patients. Transvaginal ultrasound is the preferred method for CSP diagnosis,^2^, ^12^, ^13^ which shows the positional relationship between the gestational sac, the anterior uterine muscular layer, and the bladder. At present, there are few studies on ultrasound screening for CSP patients that are suitable for expectant treatment. Some scholars speculate that myometrial thickness (MT) ≥4 mm in CSP patients could be used as an indicator for choosing expectant treatment.^14^ Therefore, this study aimed to further clarify the indication value of the lower anterior MT around the gestational sac in early pregnancy to the expectant treatment of CSP.

## Methods

The ultrasound images and pregnancy outcomes of 21 pregnant women who continued pregnancy after being diagnosed with CSP in early pregnancy were retrospectively analyzed. Patient data were collected from January 2017 to January 2020 at Changsha Hospital for Maternal and Child Health Care and Hunan Provincial Maternal and Child Health Care Hospital. Patient ages ranged from 27 to 39 years, with an average of 33.1 ± 3.1 years old. The inclusion criteria were as follows: (1) history of cesarean section; (2) diagnosis of CSP by ultrasound; (3) gestational sac with heartbeat and gestational age ≤10 weeks; and (4) hospitalization data showing pregnancy outcomes, such as a failed pregnancy or a live birth, intraoperative blood loss during cesarean section, and types of placental implantation. The exclusion criteria were as follows: (1) twin or multiple fetuses; (2) abnormal uterine morphology and uterine malformation; (3) complicated with hemorrhagic diseases; and (4) early pregnancy termination after CSP diagnosis.

The two hospitals used the same criteria to diagnose CSP. In each of the hospitals, five doctors who had practiced gynecological ultrasound for more than 2 years performed early pregnancy ultrasound. The criteria for ultrasonic diagnosis of CSP were as follows^2^, ^3^, ^15^, ^16^: (1) complete or partial gestational sac implantation or the manifestation of mixed echogenic mass in the cesarean scar on the anterior uterine wall; (2) no or partial gestational sac in the cavity of the uterine or cervical canal, closed cervical canal; (3) thin or discontinued muscular layer between the bladder wall and the gestational sac; (4) sharp edge of the gestational sac near the incision was sharp before 8 weeks of gestation, and round and blunt edge of gestational sac near the incision after 8 weeks of gestation; (5) color/power Doppler ultrasound shows high‐speed and low‐resistance blood flow signals from the cesarean scar around the gestational sac; and (6) presence or absence of heartbeat in the gestational sac.

**FIGURE 1:**
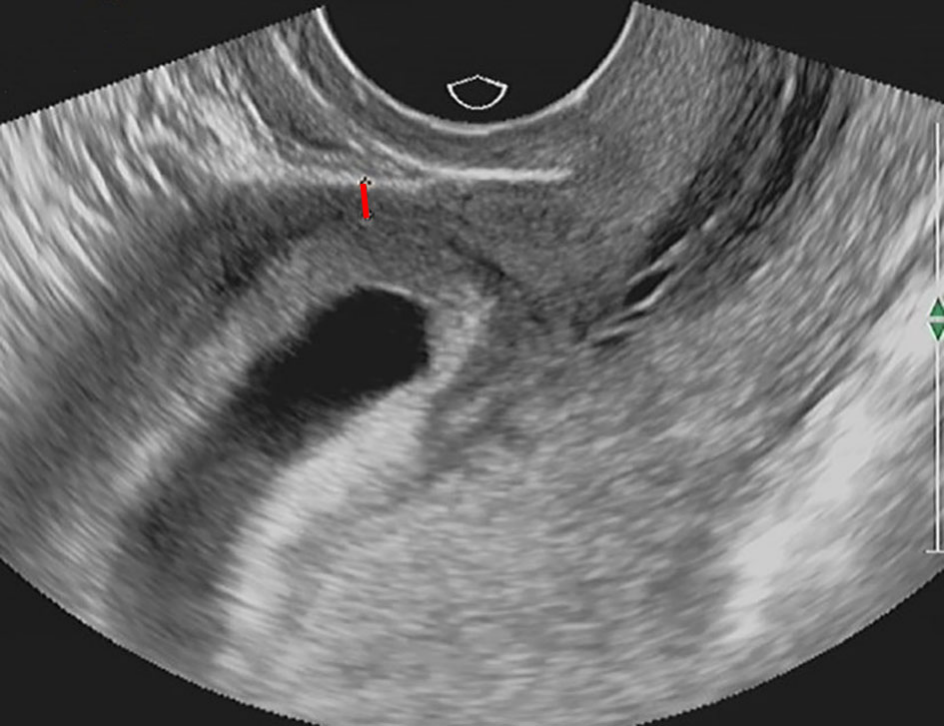
Myometrial thickness (red line) in early pregnancy was 2 mm in case no. 5 from group A, which had MAP during late pregnancy

**FIGURE 2:**
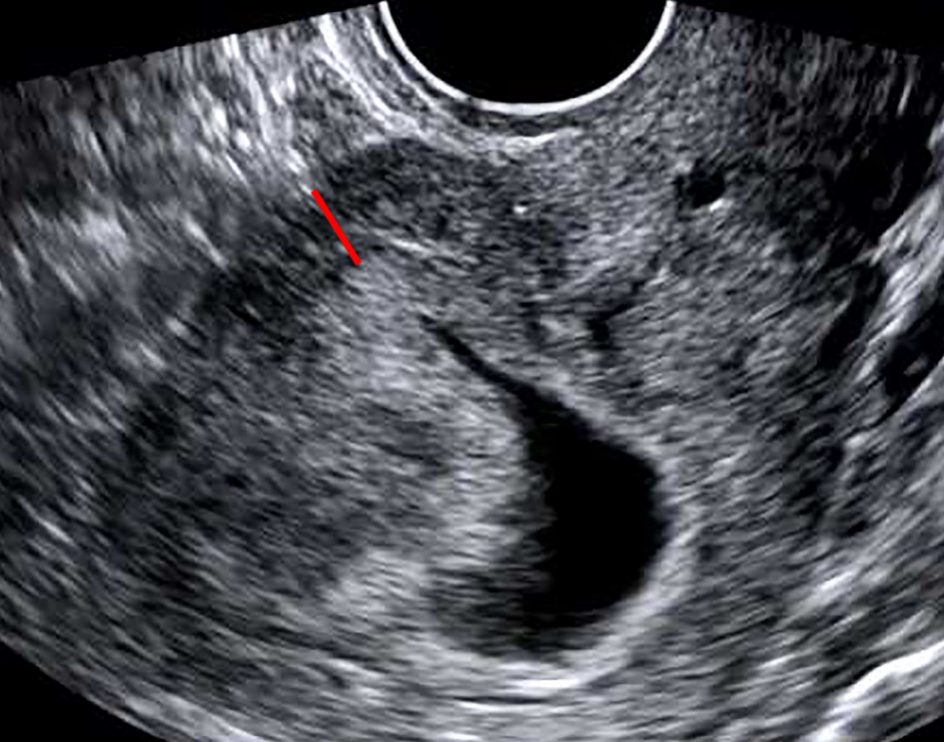
Myometrial thickness (red line) in early pregnancy was 7 mm in case no. 5 from group B, which had a normal placenta during late pregnancy

To screen for patients, ultrasound workstations in hospitals were searched for all cases diagnosed with CSP. Hospitalization was then followed, and the original case was included if the patient did not undergo pregnancy termination in the early trimester. The ultrasound images of the original case were anonymously sent to a specialist who, without knowing the clinical outcome of the cases, determined whether the case was included in the valid cases, and examined and corrected the MT measurement method in the ultrasound workstation images. After reviewing the image data from the ultrasound workstation, sagittal longitudinal sections of the uterus displayed by transvaginal ultrasound were selected. The gestational sac and lower anterior MT around the gestational sac should be clearly displayed. The thinnest lower anterior MT around the gestational sac was measured (Figures 1 and 2). Each case was measured three times, and the mean value was taken.^17^ A total 21 cases were divided into two groups. Patients with serious complications during pregnancy were assigned to group A. Severe complications included severe intraoperative hemorrhage^18^, ^19^ (intraoperative blood loss ≥1000 mL) or severe forms of MAP, such as placenta increta or placenta percreta. The remaining cases without serious complications during pregnancy were assigned to group B. Intraoperative blood loss was quantified using the volume of suction containers and weight of gauze.

All statistical analyses were performed using the statistical analysis software SPSS 25.0. Data with normal distribution were described as mean ± SD. Data with non‐normal distributions were described as *M* (*P*_25_, *P*_75_). The intra‐ and interobserver reproducibility of the measurements were evaluated using the intraclass correlation coefficient (ICC). Comparison of MT between the two groups was analyzed using the nonparametric Mann–Whitney *U* test. Statistical significance was set at *p* < 0.05. The optimal cut‐off value of MT was obtained from the receiver operating characteristics (ROC) curve analysis.

## Ethics

The Human Research Ethics Committee of Changsha Maternal and Child Health Hospital approved the data collection for this study (2021001). This study did not violate the provisions of the Declaration of Helsinki.

## Results

A total of 21 patients who met the inclusion criteria were selected for this study. There were 11 cases in group A and 10 cases in group B. Among the patients in group A, three patients with intraoperative blood loss greater than 3000 mL were admitted to the intensive care unit, and one of the three underwent hysterectomy (case 5 in group A; intraoperative blood loss, 6800 mL). Another case in group A underwent termination of pregnancy at 18 weeks due to vaginal bleeding (case 10 in group A; placenta was implanted into the bladder; intraoperative blood loss, 2000 mL). All cases in group A had a severe form of MAP. Among them, seven patients had intraoperative blood loss of more than 1000 mL, and all had blood transfusions. Therefore, the placenta of all patients with intraoperative blood loss greater than 1000 mL was MAP of severe type. In group A, there were nine (81.8%) cases with MT ≤3 mm and two (18.1%) cases with MT >3 mm. In group B, there were two (20.0%) cases with MT ≤3 mm and eight (80.0%) cases with MT >3 mm. The clinical data of all patients in group A and B are shown in Tables 1 and 2, respectively.

**TABLE 1:** Clinical data of patients in group A

Case	MT (mm)	Complications					Pregnancy outcome
		Type of placenta	BL (mL)	Hysterectomy	Blood transfusion (units)	ICU admission	
1	2.7	Placenta increta	800	No	0	No	35 + 3 weeks, CS, live fetus
2	3	Placenta increta	300	No	0	No	39 weeks, CS, live fetus
3	4.7	Placental accrete	300	No	0	No	38 + 5 weeks, CS, live fetus
4	6.7	Placenta increta	400	No	0	No	38 + 6 weeks, CS, live fetus
5	2	Placenta percreta	6800	Yes	26.3	Yes	36 + 4 weeks, hysterectomy, live fetus
6	2.2	Placenta percreta	3900	No	12.6	Yes	38 + 2 weeks, CS, live fetus
7	3	Placenta percreta	1800	No	12.6	No	36 + 4 weeks, CS, live fetus
8	5.3	Placenta increta	4200	No	20.5	Yes	37 + 1 weeks, CS, live fetus
9	2	Placenta increta	1200	No	4.6	No	35 weeks, CS, live fetus
10	3	Placenta percreta	2000	No	6.2	No	18 weeks, CS
11	1.7	Placenta percreta	800	No	0	No	34 + 6 weeks, CS, live fetus

Abbreviations: BL, blood loss; CS, cesarean delivery; ICU, intensive care unit; MT, myometrial thickness.

**TABLE 2:** Clinical data of patients in group B

Case	MT (mm)	Complications					Pregnancy outcome
		Type of placenta	BL (mL)	Hysterectomy	Blood transfusion (units)	ICU admission	
1	4.4	Normal	300	No	0	No	36 + 6 weeks, CS, live fetus
2	4.5	Normal	300	No	0	No	39 + 1 weeks, CS, live fetus
3	8.3	Placental accrete	500	No	0	No	37 + 6 weeks, CS, live fetus
4	4.7	Placental accrete	300	No	0	No	38 + 5 weeks, CS, live fetus
5	7	Normal	400	No	0	No	37 weeks, CS, live fetus
6	8.7	Normal	300	No	0	No	38 + 5 weeks, CS, live fetus
7	3.6	Normal	200	No	0	No	38 + 6 weeks, CS, live fetus
8	2.2	Normal	300	No	0	No	39 + 3 weeks, CS, live fetus
9	3.7	Placental accrete	400	No	0	No	36 weeks, CS, live fetus
10	3	Normal	400	No	0	No	39 weeks, CS, live fetus

Abbreviations: BL, blood loss; CS, cesarean delivery; ICU, intensive care unit; MT, myometrial thickness.

**FIGURE 3:**
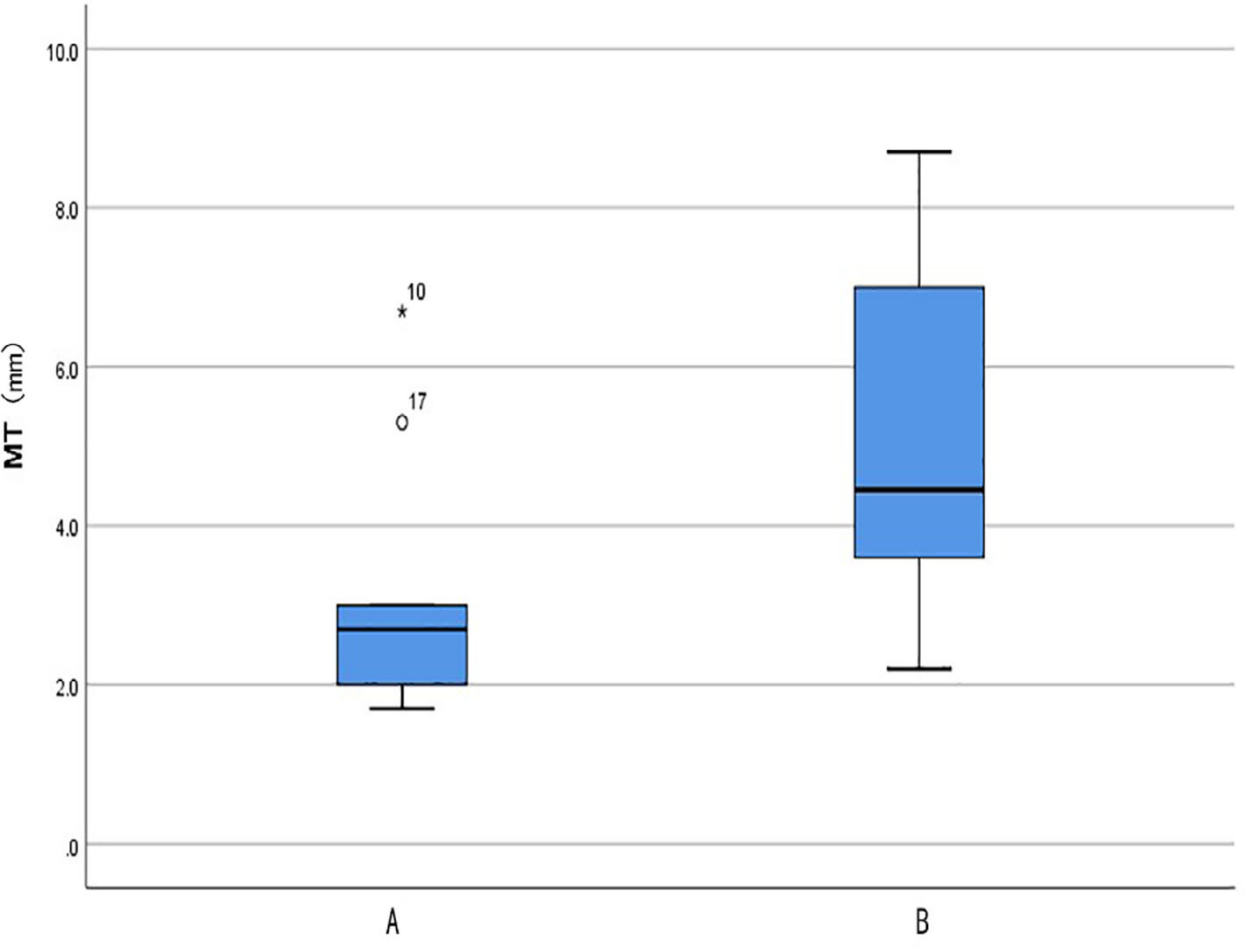
Difference of myometrial thickness (MT) between group A (with serious complications) and group B (without serious complications)

The intra‐ and interobserver agreements for MT measurement were generally good (ICC 0.86 and 0.89, respectively). In group A, the *M* (*P*_25_, *P*_75_) of MT between gestational sac and bladder was 2.70 (2.00, 3.00) mm. In group B, the *M* (*P*_25_, *P*_75_) of MT between gestational sac and bladder was 4.45 (3.45, 7.33) mm. The difference in the MT between the gestational sac and bladder in early pregnancy between groups A and B was statistically significant (*U* = 20.000, *p* = 0.013). Additionally, MT was associated with serious complications during pregnancy (Table 3, Figure 3).

**TABLE 3:** Results of the Mann–Whitney *U* test

Parameter		*N*	Mean rank	Sum of ranks	*U*	*Z*	*p*
MT	Group A	11	7.82	86.00	20.000	−2.475	0.013
	Group B	10	14.50	145.00			

Abbreviation: MT, myometrial thickness.

**FIGURE 4:**
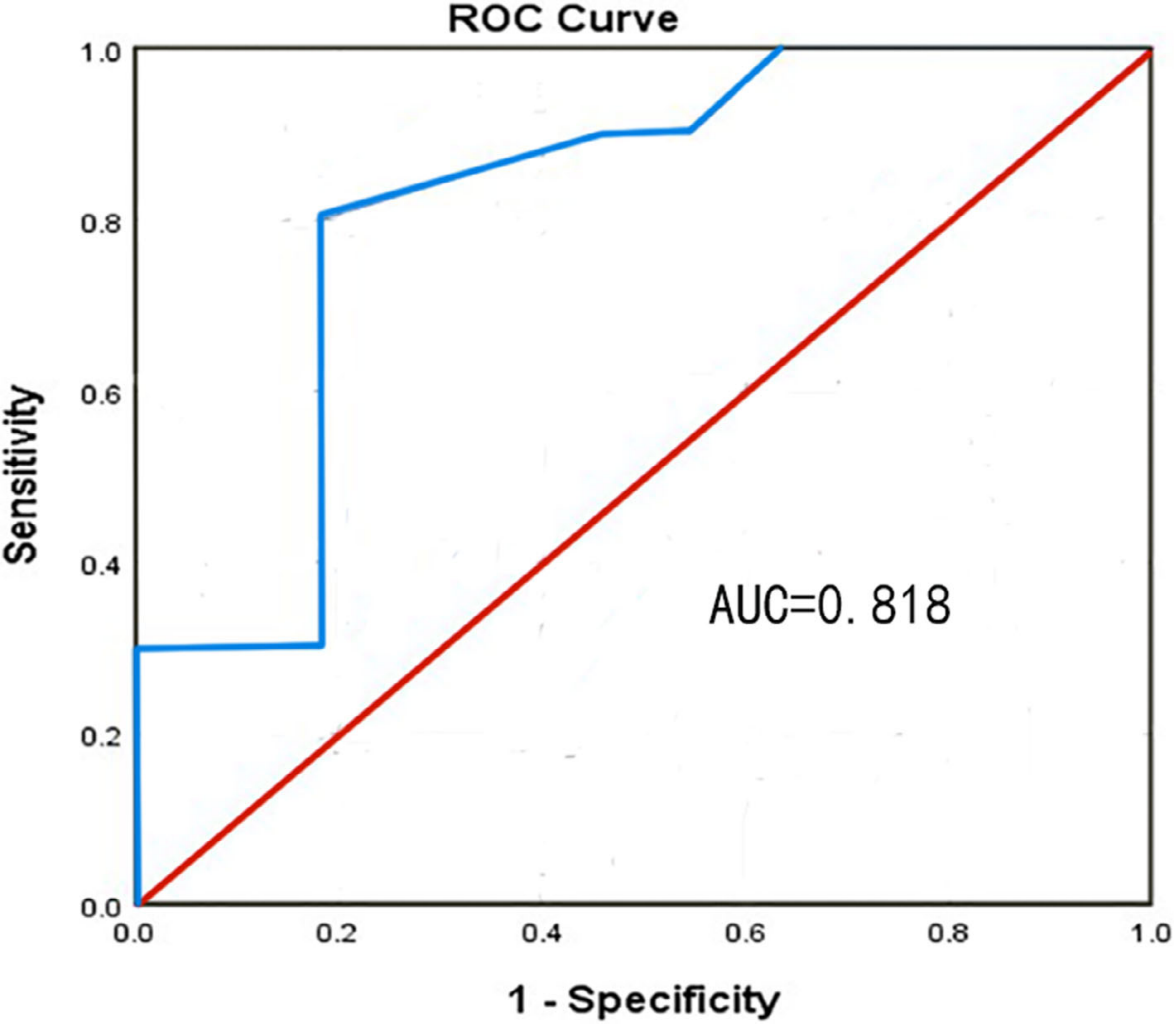
Predictive value of myometrial thickness (MT) for nonserious complications. Receiver‐operating characteristics (ROC) curves were plotted for MT

An ROC curve and Youden index were used to analyze and calculate the optimal cut‐off value of MT. The optimal cut‐off value for predicting nonsevere complications by MT was 3.3 mm (area under the curve: 0.818; 95% confidence interval: 0. 630–1.000; *p* = 0.014; maximum Youden's index: 0.618; sensitivity: 80.0%; specificity: 81.8%) (Figure 4).

## Discussion

Transvaginal ultrasonography is the preferred diagnostic method for CSP^2^, ^12^ and there are a few reports on the correlation between ultrasonic parameters and prognosis in CSP patients with expectant treatment. Some studies have suggested that the location of gestational sac implantation is related to the occurrence of serious complications. Vail et al. divided CSP into endogenous and exogenous according to the implantation location of the gestational sac.^20^ In the endogenous type, the gestational sac was partially located in the uterine cavity and partially implanted in the cesarean scar, while growing toward the uterine cavity. In the exogenous type, the gestational sac completely implanted in the cesarean scar, while growing toward the bladder. Additionally, studies showed that pregnant women with endogenous CSP may continue their pregnancy until the third trimester, but there is a risk of massive bleeding or uterine rupture. In contrast, pregnant women with exogenous CSP can suffer from massive bleeding or uterine rupture at an early stage.^19^, ^21^ The authors' hospital usually recommends early termination of pregnancy for women suspected of CSP, especially if the gestational sac is exogenous, or if there was vaginal or abdominal bleeding, abdominal pain, or any discomfort. If a CSP patient with endogenous gestational sac, who is not experiencing any discomfort and is aware of the possible serious complications, still has a strong desire to preserve pregnancy, doctors suggest that she may continue her pregnancy under close supervision, requiring the pregnant woman to review ultrasound once every 1–2 weeks to observe the direction of the growth of gestational sac before 20 weeks of gestation. Termination of pregnancy is recommended if the sac grows out of the uterus before 14 weeks of gestation. After 20 weeks of gestation, ultrasonography will be reexamined once per month. Emergency cesarean section should be performed if the mother has massive bleeding or shock symptoms. If the puerpera has a small amount of vaginal bleeding and the general condition is good, the patient should be admitted to the hospital. If the gestational age is less than 34 weeks, expectant treatment and preparation for premature delivery shall be made at the same time, such as the use of uterine inhibitors and glucocorticoids. If the gestational age is more than 34 weeks, cesarean section shall be performed after comprehensive evaluation. Elective surgery can be observed until 36 weeks of gestation if there is no vaginal bleeding during late pregnancy. All CSP patients in this retrospective analysis were of the endogenous type.

Jauniaux et al. believed that during the first trimester of pregnancy, the tip of the spiral arteries could be blocked by the extravillous trophoblast plugs in normal intrauterine pregnancies, which could prevent the free and continuous flow of maternal blood throughout the villi space of the ultimate placenta. However, for CSP patients, the permanent loss of the MT in the scar area, coupled with a reduction in spiral arteries, may result in direct contact between the anchoring villi of the primitive placenta in CSP and large‐diameter arteries of the outer uterine wall, which leads to a rapid increase in blood flow around the gestational sac in the first trimester.^22^ Therefore, it is believed that the thinner myometrial in the scar area is associated with more significant changes in the structure of the uterine wall. Moreover, the rapid increase in blood flow around the gestational sac is more significant, which may increase the occurrence of intraoperative hemorrhage.

Agten et al.^14^ believes that, for CSP patients, gestational sac implantation “on the scar” type in early pregnancy had a better prognosis than “in the niche” type. In their study, all cases with gestational sac implantation “on the scar” type and myometrium thickness ≥4 mm in early pregnancy had a good prognosis. The study suggested that expectant treatment may be considered for CSP patients with gestational sac implantation “on the scar” type and myometrium thickness ≥4 mm. In addition, Agten et al.^14^ studied the clinical outcomes of 17 patients with CSP who received expectant treatment, 12 of whom underwent hysterectomy (11 cases, placenta increta or placenta percreta; 1 case, placental accreta). The group that required hysterectomy had a MT of 0–2 mm, with an average of 1 mm, while the group that did not require hysterectomy had a MT of 4–9 mm, with an average of 5 mm. The difference in MT between the two groups was statistically significant. For CSP patients who had pregnancy termination, the study of Giampaolino et al.^23^ showed that a thinner MT and higher number of cesarean section times were associated with complications. Therefore, it is believed that the thinner the MT between the gestational sac and the bladder in CSP patients, the higher the risk of blood loss, uterine rupture, and MAP. However, there are few studies on the correlation of MT in early pregnancy with serious complications during pregnancy, such as intraoperative blood loss ≥1000 mL or with severe forms of MAP (placenta increta or placenta percreta), in CSP patients after expectant treatment.

This study demonstrates the correlation of MT between the early gestational sac and bladder and serious complications in CSP patients after expectant treatment (*U* = 20.000, *p* = 0.013). The area under the ROC curve was 0.818 (*p* = 0.014), the optimal cut‐off value was 3.3 mm, the sensitivity was 80.0%, and the specificity was 81.8%. The greater the MT, the higher the probability of nonserious complications. When the MT is >3.3 mm, the risk of serious complications, such as intraoperative blood loss ≥1000 mL or severe forms of MAP (placenta increta or placenta percreta) is relatively low. Therefore, the MT between the early gestational sac and the bladder can predict the risk of serious complications, such as severe intraoperative blood loss and severe forms of MAP after CSP expectant treatment. Moreover, MT between the gestational sac and the bladder during early pregnancy can be used as an ultrasonic parameter for expectant treatment for CSP patients. However, further studies with larger sample sizes are required.

## Conflict of interest

None declared.

## Author contributions

Liye Fu, Jinbai Huang, and Yingchun Luo contributed to the acquisition, analysis, and interpretation of the patient data. Liye Fu was a major contributor in writing the manuscript. All authors read and approved the final manuscript.

## Data Availability

The data that support the findings of this study are available from the corresponding author upon reasonable request.
